# Lithium ion conductive behavior of TiO_2_ nanotube/ionic liquid matrices

**DOI:** 10.1186/1556-276X-9-539

**Published:** 2014-10-01

**Authors:** Raman Vedarajan, Makoto Ogawa, Noriyoshi Matsumi

**Affiliations:** 1 School of Materials Science, Japan Advanced Institute of Science and Technology, 1-1 Asahidai, Nomi, Ishikawa 923-1292, Japan

**Keywords:** TiO2 nanotube, Ionic liquid, Lithium ion, Lewis acid

## Abstract

A series of TiO_2_ nanotube (TNT)/ionic liquid matrices were prepared, and their lithium ion conductive properties were studied. SEM images implied that ionic liquid was dispersed on the whole surface of TNT. Addition of TNT to ionic liquid (1-butyl-3-methylimidazolium bis(trifluoromethylsulfonyl)amide (BMImTFSA)) resulted in significant increase of ionic conductivity. Furthermore, lithium transference number was also largely enhanced due to the interaction of anion with TNT. Vogel-Fulcher-Tammann parameter showed higher carrier ion number for TNT/BMImTFSA in comparison with BMImTFSA.

## Background

In the field of ionics, a number of researchers are in search for electrolytes showing both high ionic conductivity and high selectivity for target cation transport. Also, in view of battery safety, intense researches have been carried out on solid polymer electrolytes [1-3] and non-flammable ionic liquids [4-6]. As a most common electrolyte, polyether derivatives have been widely studied for a long time with respect to their ion conductive behavior. In spite of moderate ionic conductivity of polyethers, their lithium ion transference number is generally very low at ambient temperature due to strong binding of ether oxygen to lithium cation. Their lithium ion transference number ranges only from 0.1 to 0.2 at ambient temperature. Moreover, ionic liquids which are composed of cation and anion are also unsuitable for selective target cation transport, because such component ions also migrate under potential gradient.

As a common strategy for enhancement of lithium transference number, covalent immobilization of anion structure on polymer framework [7-11] has been examined. However, this approach always resulted in decrease of ionic conductivity, due to the decrease of carrier ion number. As an alternative approach, incorporation of anion receptor in ion conductive matrices [12-21] has also been widely investigated. Particularly, boron incorporation to electrolytes is a valuable approach in which Lewis acid strength is controllable by the choice of organoboron unit. So far, a variety of organoboron polymer electrolytes were prepared by hydroboration polymerization or dehydrocoupling polymerization, and their structure–property relationship has been reported in detail.

In the present paper, titanium oxide nanotube (TNT)/ionic liquid composites were prepared, and their ion conductive properties were studied. Addition of TiO_2_ to PEO was reported to be effective in enhancing *t*_Li+_[22]. However, effect of TNT addition has not been reported so far. In the present work, addition of TNT to ionic liquid led to significant enhancement in both ionic conductivity and lithium ion transference number. As an interesting nanomaterial based on TiO_2_, synthesis of TNT was developed both by chemical method and electrochemical method (anodic oxidation) [23]. Their applications as electrodes for various energy devices have also been studied [24-28]; however, it has not been examined as an additive for lithium ion conductive electrolytes to the best of our knowledge.

## Methods

### Experimental section

The ionic liquid 1-butyl-3-methylimidazolium bis(trifluoromethylsulfonyl)amide (BMImTFSA) was synthesized by the reaction between 1-methylimidazole and 1-chlorobutane, followed by an ion-exchange reaction using lithium bis(trifluoromethylsulfonyl)amide (LiTFSA) according to literature [29]. Commercially available LiTFSA (Wako Pure Chemicals Industries Ltd., Richmond, VA, USA) was purchased and was used without further purification. Similarly, lithium sheets were purchased from Honjo Chemical Co. Ltd (Neyagawa City, Osaka, Japan). The ionic conductivity for the organic–inorganic hybrids was evaluated by complex impedance method on a Solartron 1260 impedance analyser (Solartron Analytical, Hampshire, UK) using an amplitude of 100 mV and in the frequency range of 0.1 Hz to 1 MHz. TNT was prepared by anodic oxidation method according to a literature [23]. Each sample was prepared by mixing TNT (1 mol% of BMImTFSA), LiTFSA (equimolar to BMImTFSA) and BMImTFSA and stirring the mixture for 24 h. The sample was sandwiched between two blocks of stainless steel electrodes. All samples were thoroughly dried *in vacuo* before each measurement.

The lithium ion transference number (*t*_Li+_) was measured according to the method of Evans et al. [30]. The polarization current obtained from chronoamperometry and the charge transfer resistance between the electrolyte/lithium metal electrodes observed from the impedance spectra were substituted to Evans-Vincent-Bruce equation (Equation 1), where *I*_o_ and *I*_s_, respectively, denote the initial state and steady state current. Here, *R*_o_ and *R*_s_ denote the charge transfer resistance for the initial and steady state, respectively.

(1) $$t_{\mathrm{Li}}+=\frac{I_{\left(S\right)}\left[\Delta V‒I_{o}R_{o}\right]}{I_{\left(O\right)}\left[\Delta V‒I_{S}R_{S}\right]}$$

All the operations were carried out under an argon atmosphere using identical Li electrodes using potentiostat coupled with Frequency Response Analyser (Versastat-3; Princeton Applied Research Co. Ltd., Bangkok, Thailand). Scanning Electron Microscopy (SEM) analysis was carried out on a Hitachi H-7100 (Hitachi Ltd., Chiyoda-ku, Japan).

## Results and discussion

**Figure 1:**
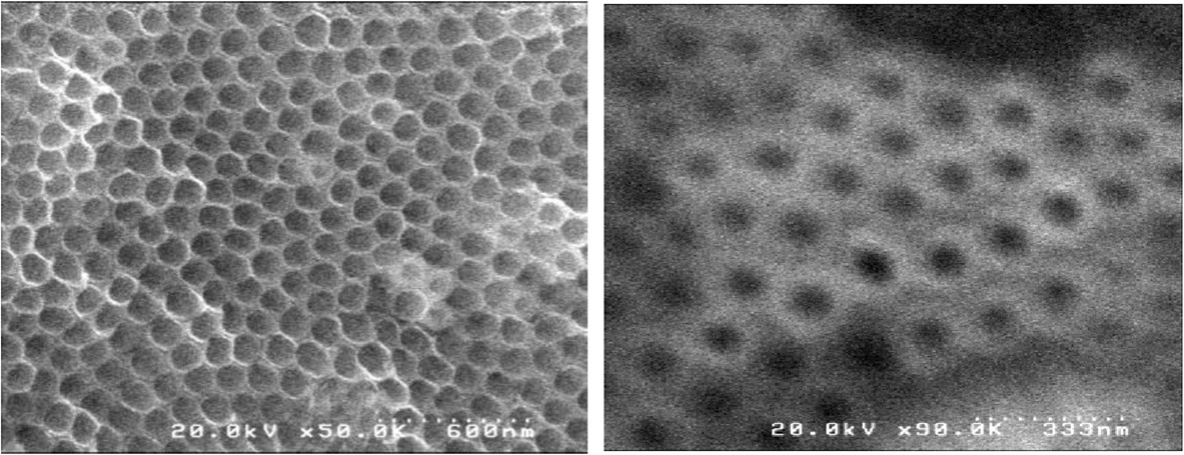
SEM images (left: TNT only, light: TNT + BMImTFSA).

Prior to electrochemical studies, the morphology of the composite was studied using SEM. SEM images for TNT (left) and TNT/ionic liquid (1-butyl-3-methylimidazolium TFSA; BMImTFSA) (right) are shown in Figure 1. As reported in literature, regular honeycomb structure of around 100 nm was clearly observed for TNT. On the other hand, the image of TNT/BMImTFSA showed lower resolution possibly due to the presence of charges on the whole TNT surface. This is supporting the formation of homogeneous TNT/BMImTFSA composite. Although TNT was slightly soluble in ionic liquid, solvation did not affect its honeycomb structure.

**Figure 2:**
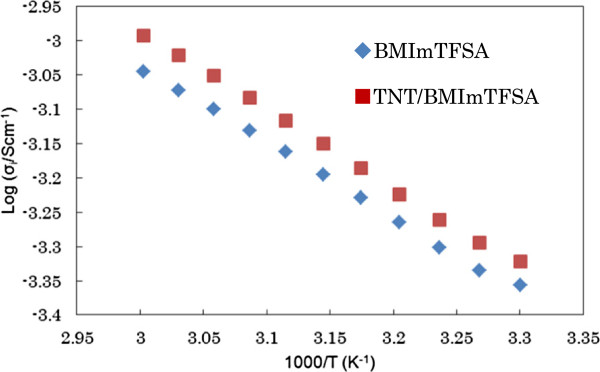
Temperature dependence of ionic conductivity for BMImTFSA and TNT/BMImTFSA.

Ion conductive properties of TNT/BMImTFSA were studied by AC impedance method after adding equimolar amount of LiTFSA to BMImTFSA. Temperature dependences of ionic conductivity for BMImTFSA and TNT/BMImTFSA are shown in Figure 2. Ionic conductivity of TNT/BMImTFSA linearly increased with raising temperature, showing that neither phase transition nor decomposition is taking place in the observed temperature range. TNT/BMImTFSA showed slightly higher ionic conductivity in comparison with BMImTFSA. The ionic conductivity of 8.3 × 10^−4^ Scm^−1^ was observed at 51°C.

Vogel-Fulcher-Tammann (VFT) fitting [31-33] was carried out to obtain further information in ion conductive behavior. After optimization of *T*_o_ (ideal glass transition temperature), successful fitting with *R*^2^ of over 0.998 was obtained. The parameters A and B stand for carrier ion numbers in the matrices and activation energy for ion transport, respectively. The VFT fitting parameters are listed in Table 1. TNT/BMImTFSA showed slightly higher activation energy for ion transport (B). However, carrier ion number (A) for TNT/BMImTFSA was also higher possibly due to promotion of salt dissociation under anion-TiO_2_ interaction, which led to significantly higher ionic conductivity for TNT/BMImTFSA.

**Table 1:** **VFT parameters for ion conductive matrices (*T***_**o**_ **= 150 K)**

	**A (Scm**^**−1 **^**K**^**1/2**^**)**	**B (K)**	***R***^**2**^	**σ at 51°C (Scm**^**−1**^**)**
BMImTFSA	1.04	759	0.9999	7.4 × 10^−4^
TNT/BMImTFSA	1.25	772	0.9983	8.3 × 10^−4^

**Figure 3:**
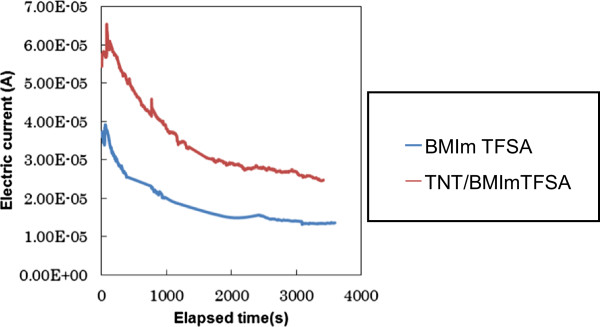
Time dependence of DC current for Li/electrolyte/Li cells at the applied voltage of 30 mV.

Lithium ion transference number of the composite was also evaluated by the method reported by Evans et al. First, time dependence of DC current was measured for Li/electrolyte/Li type cells under the applied voltage of 30 mV. After the initial decrease of DC current due to electrochemical double layer formation, gradually DC current reached steady state after 1,500 s (Figure 3). The lithium transference number was calculated using the equation reported by Evans et al. The parameters used for the calculation are listed in Table 2. TNT/BMImTFSA was found to show lithium ion transference number of 0.459 at ambient temperature, which was 1.5 times higher than that of BMImTFSA. This should also be due to efficient anion trapping effect by TNT.

**Table 2:** ***t***_**Li**_ **+ and related parameters for BMImTFSA and TNT/BMImTFSA**

	***R***_**0**_**/Ω**	***R***_**s**_**/Ω**	***I***_**0**_**/μA**	***I***_**s**_**/μA**	***t***_**Li+**_
BMImTFSA	4131	1222	36.9	13.5	0.295
TNT/BMImTFSA	28.25	173.7	65.3	25.1	0.459

*t*_Li+_(lithium ion transference number) = *I*_s_(ΔV − *I*_0_*R*_0_)/*I*_0_(ΔV − *I*_s_*R*_s_).

## Conclusions

In summary, addition of TNT to ionic liquid was found to be a fruitful method to enhance both ionic conductivity and lithium transference number via anion-TiO_2_ interaction. When interaction between anion-anion receptor is strong, enhancement of lithium transference number results in generally decreased carrier ion number and lower ionic conductivity. On the other hand, when anion-anion receptor interaction is weak, only enhancement of ionic conductivity is observed with no enhancement in lithium transference number. In the present system, moderate TNT-TFSA anion interaction led to desirable enhancement of both ionic conductivity and lithium transference number at the same time.

## Competing interests

The authors declare that they have no competing interests.

## Authors' contributions

RV carried out the morphological studies, helped MO in performing electrochemical studies, and drafted the manuscript. MO carried out electrochemical studies. NM conceived the study and helped to draft the manuscript. All authors read and approved the final manuscript.
